# Supplementary material for: A Critical View of the Application of the APEX Software (Aqueous Photochemistry of Environmentally-Occurring Xenobiotics) to Predict Photoreaction Kinetics in Surface Freshwaters
Source: Molecules. 2019 Dec 18;25(1):9. doi: 10.3390/molecules25010009 (PMC7017383; doi:10.3390/molecules25010009)
Supplement: Supplementary file 1 [file molecules-25-00009-s001.zip › 3.2.4_gcc-4.4.0.zip!/3.2.4_gcc-4.4.0/3.2.4_gcc-4.4.0/doc/octave/html/interpreter/Bug-Reporting.html]

Bug Reporting - Untitled


Next: Sending Patches,
Previous: Bug Lists,
Up: Trouble


---

### E.5 How to Report Bugs

Send bug reports for Octave to one of the addresses listed in
Bug Lists.

The fundamental principle of reporting bugs usefully is this:
**report all the facts**. If you are not sure whether to state a
fact or leave it out, state it!

Often people omit facts because they think they know what causes the
problem and they conclude that some details don't matter. Thus, you might
assume that the name of the variable you use in an example does not matter.
Well, probably it doesn't, but one cannot be sure. Perhaps the bug is a
stray memory reference which happens to fetch from the location where that
name is stored in memory; perhaps, if the name were different, the contents
of that location would fool the interpreter into doing the right thing
despite the bug. Play it safe and give a specific, complete example.

Keep in mind that the purpose of a bug report is to enable someone to
fix the bug if it is not known. Always write your bug reports on
the assumption that the bug is not known.

Sometimes people give a few sketchy facts and ask, “Does this ring a
bell?” This cannot help us fix a bug. It is better to send a complete
bug report to begin with.

Try to make your bug report self-contained. If we have to ask you for
more information, it is best if you include all the previous information
in your response, as well as the information that was missing.

To enable someone to investigate the bug, you should include all these
things:

- The version of Octave. You can get this by noting the version number
  that is printed when Octave starts, or running it with the -v
  option.- A complete input file that will reproduce the bug.

    A single statement may not be enough of an example—the bug might
    depend on other details that are missing from the single statement where
    the error finally occurs.- The command arguments you gave Octave to execute that example
      and observe the bug. To guarantee you won't omit something important,
      list all the options.

      If we were to try to guess the arguments, we would probably guess wrong
      and then we would not encounter the bug.- The type of machine you are using, and the operating system name and
        version number.- The command-line arguments you gave to the `configure` command when
          you installed the interpreter.- A complete list of any modifications you have made to the interpreter
            source.

            Be precise about these changes—show a context diff for them.- Details of any other deviations from the standard procedure for installing
              Octave.

              - A description of what behavior you observe that you believe is
                incorrect. For example, "The interpreter gets a fatal signal," or, "The
                output produced at line 208 is incorrect."

                Of course, if the bug is that the interpreter gets a fatal signal, then
                one can't miss it. But if the bug is incorrect output, we might not
                notice unless it is glaringly wrong.

                Even if the problem you experience is a fatal signal, you should still
                say so explicitly. Suppose something strange is going on, such as, your
                copy of the interpreter is out of synch, or you have encountered a bug
                in the C library on your system. Your copy might crash and the copy
                here would not. If you said to expect a crash, then when the
                interpreter here fails to crash, we would know that the bug was not
                happening. If you don't say to expect a crash, then we would not know
                whether the bug was happening. We would not be able to draw any
                conclusion from our observations.

                Often the observed symptom is incorrect output when your program is run.
                Unfortunately, this is not enough information unless the program is
                short and simple. It is very helpful if you can include an explanation
                of the expected output, and why the actual output is incorrect.- If you wish to suggest changes to the Octave source, send them as
                  context diffs. If you even discuss something in the Octave source,
                  refer to it by context, not by line number, because the line numbers in
                  the development sources probably won't match those in your sources.

Here are some things that are not necessary:

- A description of the envelope of the bug.

  Often people who encounter a bug spend a lot of time investigating which
  changes to the input file will make the bug go away and which changes
  will not affect it. Such information is usually not necessary to enable
  us to fix bugs in Octave, but if you can find a simpler example to
  report *instead* of the original one, that is a convenience.
  Errors in the output will be easier to spot, running under the debugger
  will take less time, etc. Most Octave bugs involve just one function, so
  the most straightforward way to simplify an example is to delete all the
  function definitions except the one in which the bug occurs.

  However, simplification is not vital; if you don't want to do
  this, report the bug anyway and send the entire test case you
  used.- A patch for the bug. Patches can be helpful, but if you find a bug, you
    should report it, even if you cannot send a fix for the problem.
